# Using text-mining to measure the scientific impact and legacy of ELIXIR, a distributed research infrastructure for life science data

**DOI:** 10.12688/f1000research.158264.1

**Published:** 2024-12-20

**Authors:** Francesca De Leo, Erika Balsyte, Robert Petryszak, Marilena D’Ambrosio, Chiara Bruno, Martin Cook, Ivan Mičetić, Corinne S. Martin

**Affiliations:** 1 CNR-IBIOM, Istituto di Biomembrane Bioenergetica e Biotecnologie Molecolari Consiglio Nazionale delle Ricerche, Bari, Italy, 70126, Italy; 2 ELIXIR Hub, Wellcome Genome Campus, Hinxton, England, CB10 1SD, UK; 3 Datasome, Cambridge, UK; 4 Universita degli Studi di Padova Dipartimento di Scienze Biomediche, Padova, I-35131, Italy

**Keywords:** KPI, metric, funder, literature, bioinformatics, database, resource, performance

## Abstract

**Background:**

ELIXIR is a pan-European public-funded research infrastructure dedicated to life science data. As such, it must demonstrate public value to its funders and stakeholders. We present methods to inventory research publications linked to ELIXIR that have received funding and support, as well as related citation metrics, used as performance metrics for these audiences.

**Methods:**

To overcome challenges inherent in ELIXIR’s distributed structure, and the fact that those publishing ELIXIR-supported work are typically working part-time on ELIXIR matters, a semi-automated approach, consisting of text-mining followed by manual curation, is presented. A country-level case study (ELIXIR Italy) refines and expands the methods, notably by introducing more granularity in the curation process (e.g. considering all national-level grants, examining affiliations to report publication per institute) and by additionally looking at the scientific impact of the resources developed and operated by the Italian Node of ELIXIR.

**Results:**

Overall, the methods described in this article have shown to: (1) be repeatable with acceptable levels of accuracy and consistency (notably across curators); (2) require reasonable effort in terms of curation of monthly ‘harvests’ of publications (obtained by text-mining); and (3) to be well-adapted to ELIXIR’s distributed nature.

**Conclusions:**

Concrete examples are provided of downstream uses of the inventoried publications and their citations, both for ELIXIR as a whole and for the Italian case study. Limitations of the methods are discussed, particularly the challenges associated with using an ‘Open literature’ database (Europe PMC) for the text-mining, and the constraints related to curation capacity. The methods, along with the valuable lessons learned during their development, are sufficiently generic and pragmatic enough to be readily adapted by other similar research infrastructures.

## Introduction

ELIXIR is a pan-European distributed research infrastructure dedicated to life science data, formally founded in 2013 and currently implementing its third five-year scientific programme ( ELIXIR, 2023). In 2016, it was recognised by the European Strategy Forum for Research Infrastructures (ESFRI), a forum for government officials focused on research infrastructure, as a ‘Landmark’ ( ESFRI, 2016). Landmarks are reference research infrastructures considered pillars in the European Research Area landscape, offering not only services/resources to academic research, but also supporting development and innovation. ELIXIR currently has 21 member countries, three observer countries, plus one intergovernmental organisation, the European Molecular Biology Laboratory (EMBL), which has its own country membership (overlapping in parts with that of ELIXIR).

Each ELIXIR member forms a country ‘Node’, with the European Bioinformatics Institute (EMBL-EBI) serving as the ‘European’ Node of ELIXIR. Having the Open Science principle at its heart, ELIXIR comprises over 240 research institutions across Europe, and coordinates the provision, development and discoverability of more than 500 bioinformatics resources covering databases, tools, software, interoperability standards, training, and cloud computing. These resources are financed through public funds ( Smith et al., 2020) and are typically free at the point of use, for academic and industry users alike, who can hence focus on their research rather than on the development and operation of the underlying infrastructure and its resources. ELIXIR also works to streamline and integrate national-level bioinformatics infrastructures, to strengthen national training programmes, and to bring together bioinformatics experts to create guidelines and best practices, towards ensuring the long-term sustainability of resources and infrastructure.

ELIXIR must continually demonstrate public value to its national and international funders and stakeholders more broadly to ensure their ongoing support and hence the continuity of infrastructure and resource development and operation ( Martin et al., 2021). Inventories of research publications arising from the use of the infrastructure (by others than those developing/operating it) are a common source of performance metrics requested by funders and related stakeholders ( ESFRI, 2019, OECD, 2019, European Commission, 2020). A subset of ELIXIR databases, the ELIXIR Core Data Resources ( Durinx et al., 2017), are indeed widely cited and acknowledged in thousands of research publications by life scientists across the globe ( Drysdale et al., 2020).

Around 700 bioinformaticians and related professionals across Europe form the developers and operators of the ELIXIR infrastructure and resources, and are typically working part-time on ELIXIR matters, often alongside more conventional research activities. These professionals usually have research profiles and, through their daily work on ELIXIR matters, actively contribute to the generation and dissemination of new technical and scientific knowledge linked to the development and operation of the ELIXIR infrastructure and its resources. These efforts are enabled (in part or fully) by a range of funding sources, such as ELIXIR’s internally-funded projects (also referred to as ‘Commissioned Services’), national-level financing, and grant funding from the European Union and other international funders awarded in the name of ELIXIR. In line with the Open Science principles, this new knowledge is shared openly via ELIXIR-supported research publications (peer-reviewed, preprints), which collectively represent an additional source of performance metrics of ELIXIR’s scientific legacy as a research infrastructure.

The purpose of this article is to present the methods that have been developed and implemented to inventory research publications that have received funding and support linked to ELIXIR, as well as related citation metrics. The methods are presented for ELIXIR as a whole (lightweight, considering all Nodes) and for a particular country Node, ELIXIR Italy, itself also of distributed nature. For the latter, the methods were refined thanks to greater curation capacity at the level of the Node, and also expanded to include the inventorying of research publications citing resources developed and operated by the Node. This exercise is very informative of the Node’s scientific impact and legacy, yet it would not easily scale for the whole distributed infrastructure (in terms of curation capacity at the ELIXIR Hub, the infrastructure’s coordinating secretariat) since the infrastructure operates hundreds of resources under the ELIXIR umbrella.

A semi-automated approach, consisting of text-mining followed by manual curation, is presented, with the Italian case study going further so as to better reflect the needs and expectations of national-level funders and other (internal and external) stakeholders of the Node. Of note, the guiding principle in developing these methods was that they would not require extensive technical nor programming knowledge to be applied once in place - as a result, the methods do not involve any modelling (e.g. with training and test sets), nor do they claim to be rigorously accurate. It is hoped that the steps taken, and lessons learned (both ELIXIR-wide and at the scale of a country Node), in arriving at operational methods in the context of distributed organisational settings will be useful to other such research infrastructures and organisations, in the life sciences and other disciplines.

## Methods

### Lightweight methods for the ELIXIR infrastructure as a whole (all Nodes)

**Main challenges**

Two intrinsic challenges that the lightweight methods needed to overcome were (1) that those publishing ELIXIR-supported work are typically working part-time on ELIXIR matters, and (2) the scale of ELIXIR’s “people infrastructure” (more than 700 specialists across Europe, showing a level of staff turnover). The latter itself represented a significant barrier to manual reporting of publications. As for the former, traditional bibliometrics/scientometrics approaches centred on researchers’ unique identification numbers (e.g., ORCID, ResearcherID/Publons), and/or institutional affiliations, were deemed inadequate for identifying research works (funded and/or) supported by ELIXIR, as these approaches would result in significant amounts of ‘false positives’, i.e. publications not supported by ELIXIR. To overcome these two challenges, the lightweight methods were designed to (1) detect, through text-mining within an Open research literature database ( EuropePMC, Rosonovski et al., 2024), publications (peer-reviewed and preprints) in which there were acknowledgments of the support and/or funding received from ELIXIR, (2) followed by manual curation for increased accuracy.

**Sources of ELIXIR support**

For the ELIXIR infrastructure as a whole (all Nodes), two main streams of funding and/or support were considered: (1) ELIXIR’s internally-funded projects, i.e. Commissioned Services comprising Implementation Studies, Staff Exchange, Knowledge Exchanges and Industry days, and Travel Grants, where funding eligibility is restricted to institutes part of the ELIXIR membership, and (2) grant funding from international funders such as the European Union (notably under the Research Infrastructures Work Programmes), in which several ELIXIR institutes are beneficiaries and the purpose of the grant is to develop and/or operate the ELIXIR infrastructure.

**Searching for relevant publications using text-mining**

EuropePMC’s Application Programming Interface (API) was used to search for relevant publications (technical documentation), as well as their citations in the Open literature (technical documentation), considering all years for which ELIXIR existed, even as a concept ( *circa* 2011), with a cut-off date of December 2023 (for the purpose of this article). Search configuration parameters ( Table 1), i.e. ‘search terms’ for short, comprised unique grant identification numbers and names, names and short codes of countries that are members of ELIXIR (e.g. ELIXIR Norway, ELIXIR NO), as well as “boiler-plate” text strings relating to ELIXIR support and/or funding including for ‘signature’ events (e.g. the ELIXIR-convened Biohackathon Europe) and technical workshops/meetings. For use in EuropePMC’s API, the search terms (query parameters) were first URL encoded using the online utility URL Encode/Decode, provided free, courtesy of Dan’s Tools: for example, the EU funded project ELIXIR-CONVERGE would be encoded as “ELIXIR%2DCONVERGE”. Of note, EuropePMC is case insensitive, meaning that it is not necessary to provide search terms in both lower/upper cases. For the lightweight methods, acknowledgement of the use of the infrastructure and its resources was deemed beyond scope due to the mismatch between curation capacity at the ELIXIR Hub and the several hundreds of resources operated under the ELIXIR umbrella.

**Table 1.** Search configuration for lightweight methods across the ELIXIR infrastructure as a whole.

Type	Search terms (examples)
Unique grant identification numbers	730941; 654248; 825575; 676559; 824087; 739563; etc
Grant names	ELIXIR-EXCELERATE; ELIXIR-CONVERGE; EOSC life; FAIRplus; etc
Names and short codes of countries that are members of ELIXIR	ELIXIR BE; ELIXIR Belgium; Belgian ELIXIR Node; ELIXIR GR; ELIXIR Greece; Greek ELIXIR Node; Hellenic ELIXIR Node; EMBL-EBI; etc
“Boiler-plate” text strings	This work was funded by ELIXIR, the research infrastructure for life science data; European life sciences Infrastructure; ELIXIR Implementation Study; ELIXIR Implementation Studies; ELIXIR Commissioned Service; ELIXIR Travel Grant; ELIXIR Staff Exchange; ELIXIR BioHackathon; Biohackathon Europe
Generic search terms	ELIXIR Europe; ELIXIR Node; funded by ELIXIR; funded partially by ELIXIR; supported by ELIXIR; ELIXIR Core funding; funding from ELIXIR

An initial set of search terms were entered in a Google Sheet, and retrieved from there via a specific Google Sheets API. This allowed for the subsequent dynamic configuration of the search queries when new grants were awarded to ELIXIR. The Google Sheet interacted with the EuropePMC API based on the retrieved search configuration, and the text-mining process resulted in the generation of tab-delimited text files listing potentially relevant publications comprising a mix of ELIXIR-related works (‘true positives’) and false positives.

**Manual curation and search optimisation**

Manual identification of false positives was carried out using the tab-delimited text files, one column of which collated the triggers relevant to each returned publication. Triggers represented the search terms that had been detected in the publications and were found to guide curation very effectively. False positives were blacklisted for future searches. To ensure consistency, reproducibility and the lowest subjectivity possible in curation decisions, curation efforts were carried out by a small team of two ELIXIR Hub staff who kept a record of difficult and/or unclear cases, and of the decisions taken. They were supported on an ad-hoc basis by additional ELIXIR Hub staff who had more extensive knowledge and understanding of ELIXIR’s activities in certain thematic and technical areas.

During the initial development of the methods, iterative searches and manual curation rounds were undertaken to test and refine the set of search terms, and to identify which sections of EuropePMC entries (for each publication) were the most informative to target by text-mining - these were found to be the title, first author, funding statement, and acknowledgments. The initial text-mining runs naturally returned large backlogs of potentially relevant publications since they covered the years 2011 to 2019: although this made the curation effort significant at the time, it also provided a rich list of results to optimise the searches, notably to identify which sections ( Table 2) of the publications ought to be extracted to the tab-delimited text files used during manual curation and any downstream visualisations.

**Table 2.** Extracted publication sections from EuropePMC, used for manual curation and downstream visualisations.

Sections of the publications
Harvest month
Triggers identified
Unique EuropePMC identification number
Digital Object Identifier (DOI)
Title
Journal name
Publication year
First author name
First author affiliation
Last author name
Last author affiliation
Funding statement
Acknowledgements

A set of known ELIXIR-supported publications was useful for fine-tuning the approach, as they would be expected to be returned by the searches. If this was not the case, underlying reasons would be investigated and, where possible, solutions found - if no solution was found, these publications were added (via their unique EuropePMC identification number) to a whitelist that would be pulled to the tab-delimited text file, regardless. Later on, once the methods were operational ( *circa* early 2020), the text-mining “harvests” (i.e. searches) followed by curation ended up being carried out on a monthly basis, to identify recently published and/or recently indexed publications, based on the latest set of search terms.

Search terms returning very high numbers of false positives for very few true positives were gradually excluded from searches. This was for instance the case of “ELIXIR” as a standalone term (misunderstood for the word “elixir”), “implementation study” (rather than the more precise “ELIXIR implementation study”), and the project name “BY-COVID” (incorrectly read as the very common text string “[infected] **by COVID**-19”). This was to strike a balance between accuracy (i.e. not missing any relevant publication) and overwhelming curators. Rather, it was deemed more useful to arrive at manageable monthly text-mining harvests and curation workloads moving forwards (approximately 3 to 4 hours per monthly harvest), since the intention was to arrive at operational performance metrics that could be monitored in the long-term, rather than a very accurate one-off exercise.

### Case study: refined and expanded methods focusing on a single Node (ELIXIR Italy)

Unless otherwise specified, the methods for the Italian case study are identical to the lightweight one described above (for ELIXIR as a whole).

**Sources of ELIXIR Italy support**

Building on the experience of the ELIXIR Hub effort, the Italian case study began at the end of 2022 and was focused on the period January 2015 (the year when Italy became an ELIXIR member country) to December 2023 (cut-off date for the purpose of this article). The main three sources of funding and/or support considered in the Italian case study were (1) those ELIXIR internally funded projects in which one or more of the institutes of the Italian Node were involved, (2) ditto for grant funding from international funders, and (3) national-level grants awarded to the Node to develop and/or operate its infrastructure and resources.

**Searching for relevant publications using text-mining**

For the Italian case study, the ‘ELIXIR as a whole’ set of search terms was refined to remove terms not relevant to the Node, e.g. if the Italian Node was not a beneficiary of a given grant. Additional search terms were introduced (e.g. national-level grants) along with other terms (e.g. Italian Node of ELIXIR, often used instead of ELIXIR-IT or ELIXIR IIB) that were known to be mentioned in publications acknowledging support and/or funding by ELIXIR Italy ( Table 3).

**Table 3.** Search configuration for methods focused on a single country Node (Italy).

Type	Search terms (all)
Unique grant identification numbers	951724; 101046203; PIR01_00017; 871075; IR0000010; 824087; 857650; 825575; 101057388; 676559; 101003551; 101017549; 653549; 956137; 862658; 101016167; 778247; 823886; 654008; 634486; 952334; 101081813
Grant names	BEYOND 1M GENOME; BY-COVID; CnrBiomics; ELIXIR-Converge; ELIXIRNextGenIT; EOSC-Life; EOSC-Pillar; European Join Programme On Rare Diseases; EuroScienceGateway; EXCELERATE; Exscalate4Cov; Genomed4All; INDIGO DataCloud; IDPfun; NewTechAqua
Generic search terms	ELIXIR IIB; ELIXIR-IIB; ELIXIR ITA; elixir iib; elixir-iib; Italian Node of ELIXIR; ELIXIR-IT

The major expansion of the methods followed Drysdale et al. (2020) and their focus on citations of ‘key articles’ describing the Node’s resources. This additional search effort was approached by using the Digital Object Identifier (DOI) of these ‘key articles’ to collate citation data and use this as proxy for the scientific impact of the Node’s resources. For example, the resource PatSearch was first described in Pesole et al. (2000) and an improved version of it was described in Grillo et al. (2003) - these are considered the resource’s ‘key articles’. The list of ELIXIR Italy resources was based on the 2023 version of the Node’s Service Delivery Plan under the ELIXIR umbrella, and the DOIs were sourced from the bio.tools registry ( Ison et al., 2019). For the 65 resources developed and operated by ELIXIR Italy in 2023, a total of 124 DOIs were hence collated, representing the ‘key articles’ describing initial and improved versions of these resources.

**Manual curation and search optimisation**

Curation in the context of the case study was a very similar process to that described above but carried out by ELIXIR Italy staff with knowledge of the Node’s activities. Author affiliations were additionally scrutinised and encoded to obtain a more granular view of dissemination efforts and scientific impact at the level of the Node’s institutes (30 in total at the time of writing). Sources of false positives were like that described above, e.g. project names such as “ORCHESTRA” (understood as the word “orchestra” or other projects called as such). As above, a balance was struck between accuracy and curation capacity by excluding problematic search terms (those returning high levels of false positives).

## Results

### ELIXIR infrastructure as a whole (all Nodes)

**Figure 1.**
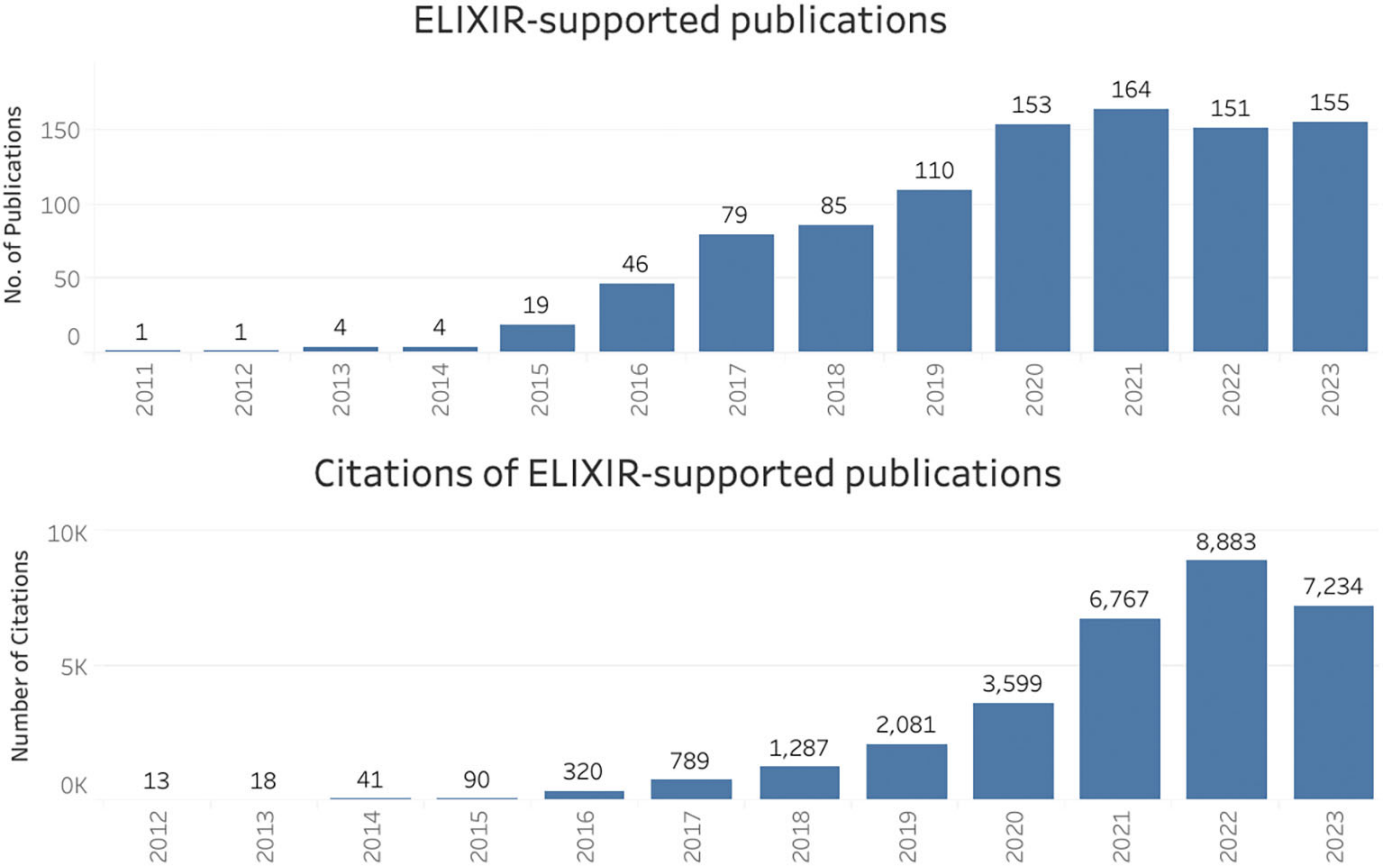
Publications and citations supported by ELIXIR (2011–2023) in Open literature (EuropePMC).

For the period 2011 to 2023, 972 publications supported by ELIXIR (as a whole) were identified, and these were collectively cited over 31,000 times ( Figure 1). The curated inventory of publications and their citations in the Open literature were used to create visualisations in Tableau software, which were themselves featured on ELIXIR’s impact dashboard, among other indicators relating to ELIXIR. On this dashboard, the page showing ELIXIR’s scientific legacy as a research infrastructure received nearly 600 unique visitors from over 40 countries in just 12 months (December 2022 to November 2023).

### Case study: ELIXIR Italy

**Figure 2.**
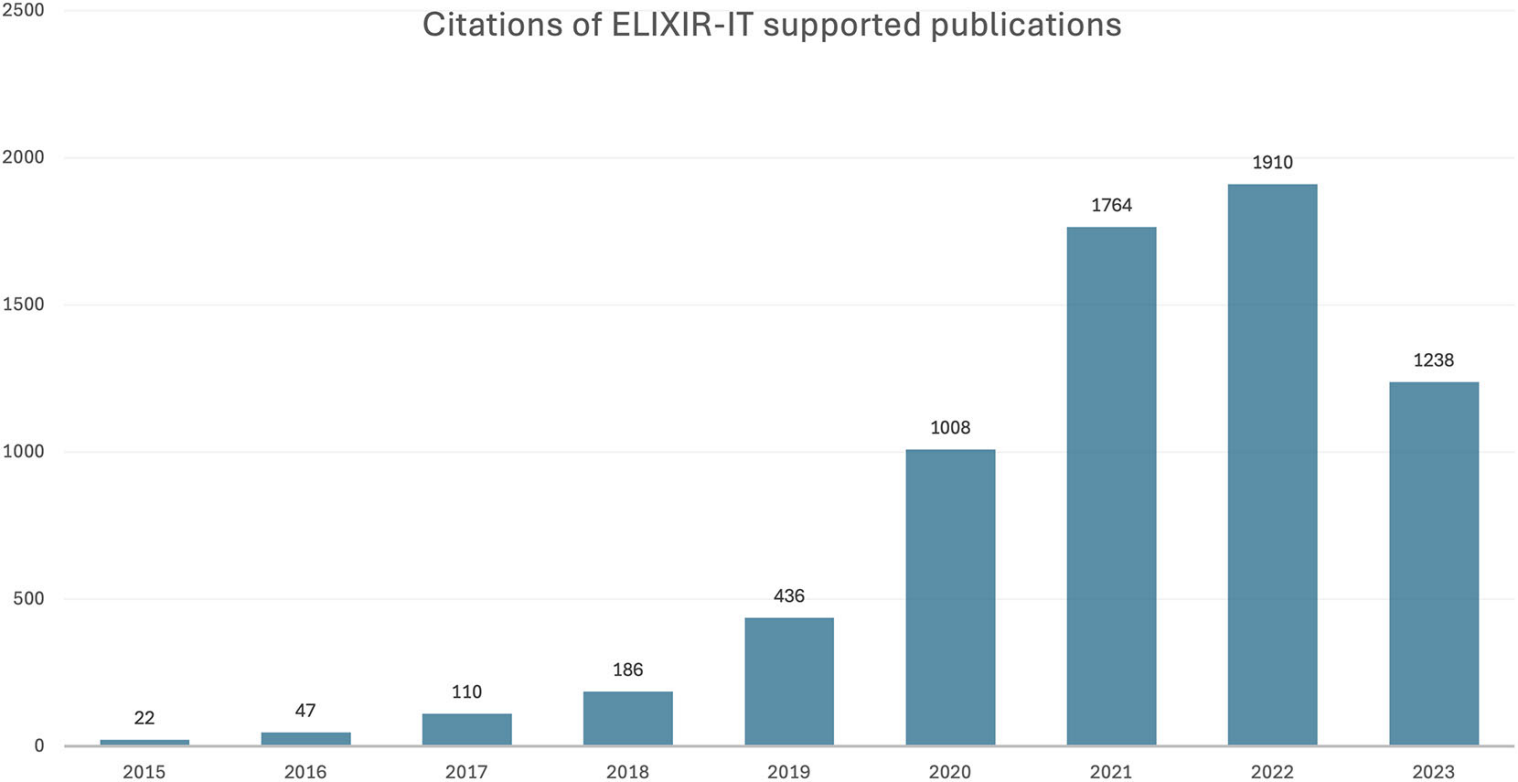
Publications and citations supported by ELIXIR Italy (2011–2023) in Open literature (EuropePMC).

**Figure 3.**
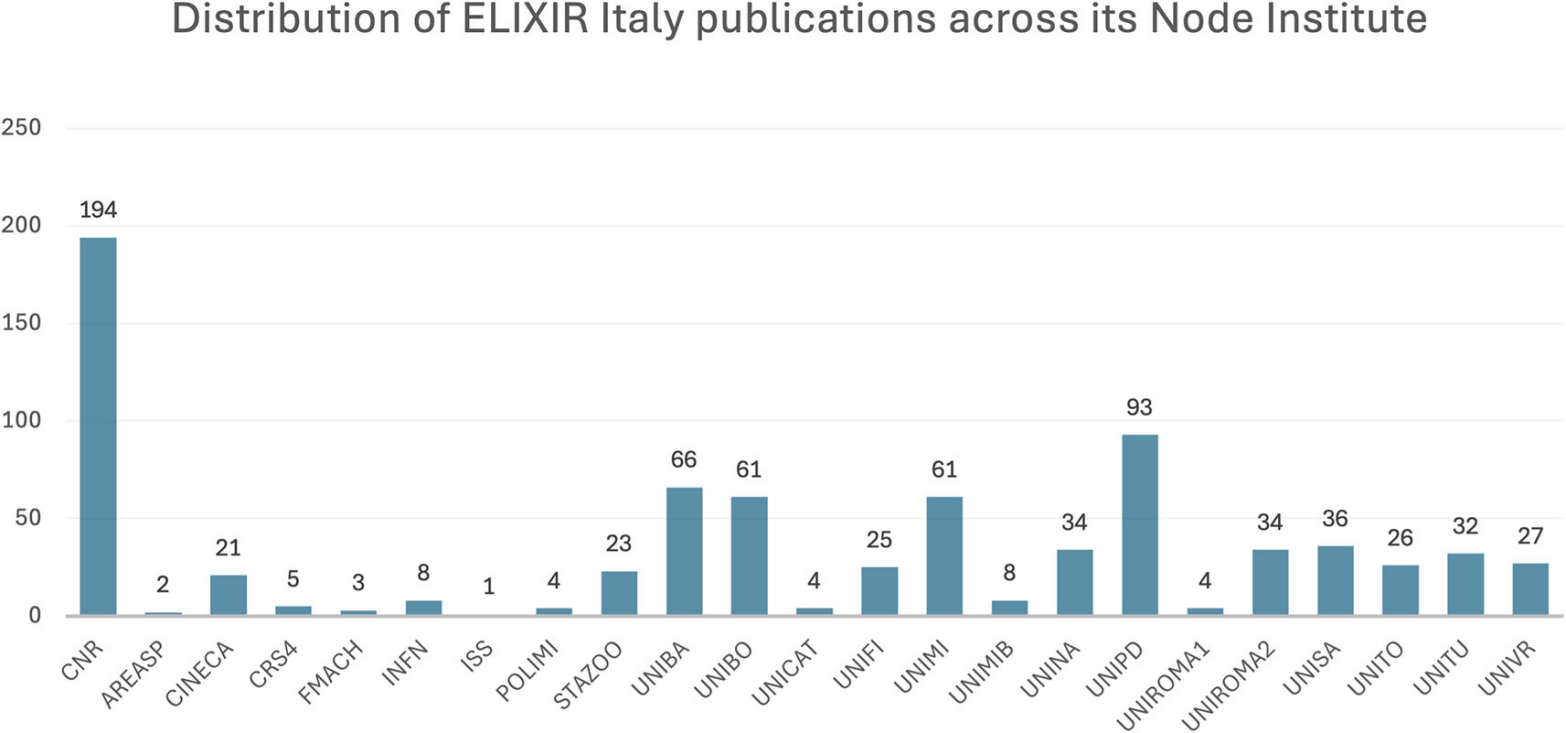
Distribution of ELIXIR Italy publications across its Node institutes. Acronyms: see https://elixir-italy.org/about/members/.

For the period 2015 to 2023, 408 publications supported by ELIXIR Italy were identified, which were collectively cited almost 5,000 times ( Figure 2). The distribution of publications across Node institutes ( Figure 3) highlights the disproportionate contribution of the National Research Council (NRC), though unsurprising given its role as the coordinating institute of ELIXIR Italy. Associated visualisations can be accessed on ELIXIR Italy’s impact dashboard which, for a 24 month period (January 2022 to December 2023), received over 5,000 unique visitors from 10 countries. In terms of the impact of resources developed and operated by ELIXIR Italy ( Table 4), citations of their ‘key articles’ amounted to 8,700, across the five ELIXIR Platforms, which are the technical domains of implementation of the infrastructure.

**Table 4.** Citations of ELIXIR Italy's resources via DOIs of their ‘key (descriptive) articles’.

Year	2015	2016	2017	2018	2019	2020	2021	2022	2023
Tools Platform	327	424	476	450	456	571	713	805	703
Data Platform	304	311	289	342	339	398	557	570	461
Compute Platform	14	12	10	4	15	15	26	19	19
Interoperability Platform	0	0	0	1	2	0	6	32	29
Total citations per year	645	747	775	797	812	984	1,302	1,426	1,212
Total citations	8,700								

## Discussion

In almost four years of implementation, the methods have been found to be adequately performant at identifying publications supported by ELIXIR, if the relevant triggers were present (and correctly spelled) in the publications. Since the inception (in 2020) of a dedicated “How to acknowledge ELIXIR funding and support” page on the ELIXIR website (with the analogous one on the ELIXIR Italy site, bottom part), curators have noticed clear improvements in how ELIXIR’s contribution is acknowledged.

The upward trend in citation numbers for both the lightweight and case study methods suggested long-term scientific impact of these publications, a reminder that impact is slow to develop. It is not just citations that take time to appear: large-scale projects such as ELIXIR-EXCELERATE which involved 47 partners from 17 countries ( Harrow et al., 2021; €19 million, 2014-2019) were found to be still acknowledged in publications years after their completions, This is not solely due to delayed indexing but also to the enduring legacy of their support for research activities within teams connected to the project.

Overall, the methods described in this article have shown to: (1) be repeatable with acceptable levels of accuracy and consistency (notably across curators); (2) require reasonable effort in terms of curation of monthly harvests (in contrast to running an ELIXIR wide manual reporting process); and (3) to be well-adapted to ELIXIR’s distributed nature (including at Node-level in the Italian case study).

While the text-mining component can be operated with minimal ELIXIR expertise, it is true that the curation component requires a deeper awareness and understanding of the organisation’s activities. One example is when ELIXIR was involved in large “cluster” grants (e.g. EOSC-life ) in which many life science research infrastructures were also funded, meaning that not all publications acknowledging EOSC-life’s grant would be relevant to ELIXIR, despite ELIXIR coordinating the project.

The Italian case study highlighted the usefulness of national-level granularity in the search terms. Whilst it is true that the lightweight ‘ELIXIR as a whole’ methods do include a few national-level grants in its search terms, this is far from being fully implemented. Their inclusion was exploratory at best and, based on this experience, there is insufficient appetite at the ELIXIR Hub to fully implement national-level search terms due to capacity constraints in terms of curation and limited knowledge of ELIXIR activities at Node level.

### Limitations of the methods linked to using an ‘Open literature’ database (EuropePMC)

One main limitation of the methods is linked to EuropePMC being used as the central source of publication data - only the publications indexed in this literature database, focused on life sciences, can be returned by the searches. At the time of writing, specialised journals such as Data Science Journal and Data Intelligence were not yet indexed due to perceived low demand of these in the user base of EuropePMC. However, based on user feedback, EuropePMC started indexing BioHackrXiv in 2021, especially as it is the recommended preprint server for outcomes of the ELIXIR-convened Biohackathon Europe series ( Castro et al., 2021).

Although research funders are increasingly requesting that the work they fund is openly accessible, it was found that certain ELIXIR-relevant publications were behind paywalls. This meant that EuropePMC was not able to fully “ingest” the text of certain sections of the source text, e.g. funding statement, which made text-mining efforts fail, despite the publication having a presence in EuropePMC and the right trigger being present. This was manually solved on an ad-hoc basis by whitelisting these publications using their unique EuropePMC identification number. Additionally, this method can effectively identify ELIXIR-related publications that are non-compliant with open access policies and funder requirements.

Looking forward, EuropePMC continues to work closely with scientific publishers to agree and implement standards so that ingestion of information from the publishers’ sites to EuropePMC is more effective. This is an on-going and quite a considerable task, and additional sources of text-mining failures included conflicts in metadata, embargoed works in paywalled journals, as well as citations being limited to the Open literature, the latter leading to underestimated scientific impact.

### Uses of the curated inventories of publications as source of performance metrics

The intention behind this work was to arrive at operational performance metrics that could be easily monitored in the long-term, in contrast to a rigorous and hence heavy-duty process requiring significant resources, likely to be carried out once or twice at best. The curated inventories of publications, including their citations, have so far been used as input into ELIXIR’s annual reports, ELIXIR newsletters and social media posts (under the heading “Recommended reading”), technical reporting to funders, as well as ELIXIR’s monitoring report to ESFRI and a growing number of requests for information from national funders, such as Sweden ( Swedish Research Council, 2021), Finland ( Academy of Finland, 2023) and the Netherlands ( NWO, 2023).

Similar to ELIXIR, ELIXIR Italy has used the curated inventories of publications and their citations for a range of internal and external monitoring activities, as required by various stakeholders, for example periodic Node reviews and presentations for ELIXIR’s Board of funders, Scientific Advisory Board and Industry Advisory Committee. The information has also been used to report to ELIXIR Italy’s own Scientific Advisory Board, as well as for the Node’s general assembly and monthly newsletters. Finally, the information has been used to report to ELIXIR’s Italy’s main funder, the Italian Ministry for Universities and Research (MUR)), notably as part of evaluation processes and of national-level grant funding for research infrastructures.

## Conclusions

As illustrated by the Italian case study, these methods represent the first step of potentially many exciting downstream analyses using the curated inventories as starting materials. Their citations would shed light on the impact of these works, through their use by others. Yet these inventories alone have proved to be useful already, in terms of communicating performance and impact of both ELIXIR activities (as a whole and for a country Node) and the use of a Node’s resources. The Italian case study also demonstrated the value of knowledge sharing within an organisation, in the spirit of Open Science, with the country Node leveraging the experience of the ELIXIR Hub, building on and enhancing it.

The methods’ main strength lies in its relative ease of implementation, especially in the context of a distributed life science research infrastructure staffed with highly qualified and research-active personnel: many will have the programming skills required to operationalise the methods, whilst no such technical skill is required for the curation part of the methods. If not, external assistance can be sought in the form of consulting, to get the approach off the ground. Another strength of the methods is its versatility as new funding streams become available and resources are further developed.

The methods, along with the valuable lessons learned during their development, are generic and pragmatic enough to be readily adapted by other similar research infrastructures. This extends its potential application beyond life sciences to encompass physical sciences, social sciences, and the humanities.

### Ethical considerations

Ethical approval and consent were not required.

## Data Availability

EuropePMC ( Rosonovski et al., 2024; https://europepmc.org/), an Open research literature database, was used as the source for the underlying data (publications in the life sciences) used in the methods described in the article. The extended data created as a result of applying the methods are curated inventories of publications that have received funding and support from ELIXIR. They have been deposited in Zenodo ( Martin et al., 2024; https://doi.org/10.5281/zenodo.14136249) under a Creative Commons Attribution 4.0 International license, so that others can access them for illustrative and verification purposes. N/A
