# Supplementary material for: Using text-mining to measure the scientific impact and legacy of ELIXIR, a distributed research infrastructure for life science data
Source: F1000Res. 2025 Apr 22;13:ELIXIR-1547. Originally published 2024 Dec 20. [Version 3] doi: 10.12688/f1000research.158264.3 (PMC12498510; doi:10.12688/f1000research.158264.3)
Supplement: Peer review history

# Reviewer report: Reviewer response for version 3

**DOI:** 10.5256/f1000research.180668.r380723

**Published:** 2025-05-29

**Authors:** Alfonso Valencia

**Affiliations:** 1 Barcelona Supercomputing Center., Barcelona, Spain

First, let me congratulate the authors for addressing this challenging and to some extend nasty task full of details. 

 I have a number of concerns related with the technical part, the presentation of the results and the discussion. 

 Overall, my assessment is that the authors have an interesting collection of results that are only partially exposed and very superficially analysed. Given that the hard work has been done it is a pity not to complete it with a relevant analysis answering what the title announces: measuring the impact and legacy of ELIXIR (for example, after reading the paper, I still do not know if the answer is a lot or little impact or where has ELIXIR made more impact)

 1.- The usual - and recommended - practice is to develop a  handbook with the details steps for the curation (Annotation Guideline). A handbook describing in detail the steps for the curation of the documents and the construction of the corpus, that annotators have to follow.

 The annotation guidelines are necessary for the alignment of the  annotators, to decrease the inter-annotator error, for the validation of the procedures and are useful for other to be able to do a similar exercise in the future. An example (in spanish: https://zenodo.org/records/6477407).

 In many senses, they are the most important legacy of any annotation process. This publication comes halfway to provide the information for the annotation handbook.

 2.- Regarding manual curation. where are the numbers of raw identifications and the number of articles rejected and  for what reasons? 

 This information will  be very interesting to better understand how effective the text mining was and what are the main source of problems.

 3.- The authors discuss the limitations of the study including the access to non-open literature. This is true, but it is also true that the grants are quoted, at least in part, in the Medline entries. Is this information used? or only from full open paper in EuroPMC? and if not, why not?

 4.- Table 2 with the sources of publications is important, but it does not give the numerical information on how many articles were extracted with each query and what was the overlap ?  This information will be important to understand the influence of the different sources and to plan future experiments. 

 5.- I find difficult to understand the section dedicated to the retrieval of "key articles" and how articles quoting an ELIXIR resource were processed to identify contribution of ELIXIR-IT. Where the articles quoting key resources considered part of the ELIXIR-IT contribution even if they did not acknowledge a specific grant?

 6.- Related with the previous point. The case study is interesting but I find the analysis of the data insufficient. 

 Apparently, a quarter of the ELIXIR citations are from publications "solely" by ELIXIR.-IT? this seems to be too many, and likely many of the ELIXIR publications are derived from collaborations between ELIXIR-IT and other ELIXIR nodes. 

 What is the overlap? how many are unique ELIXIR-IT publications? and to what do they correspond (areas of elixir? Core tools produce in Italy - detailing what is given  in Fig 5?)

 7.-The problem with the umbrella institutions is well known. In other cases, efforts have been made to disambiguate the affiliations to increase granularity by, for example, using the other addressed in the same paper.

 8.- Fig 2. legend is publications and citations but only citations are shown.

 9.- I do not understand how table 5 was generated. HOw the articles (how many?) dedicated to the core resources are assigned to the platforms? How many papers and citations have each one of them?

 10.- Table 5. Will be correct to say that the Italian core resources were quoted 1426 times in 2022 and that in total publications of ELIXIR-IT were quoted 1910 times this year?I If this is the case, it will be important to discuss this point: the main source of impact of ELIXIR are the core resources and not the work in the grants (75% of the citations) right?  Is this the case of ELIXIR or only for ELIXIR-IT?

 11.- After making a selection of papers by grants, and even if not all the papers quote the corresponding grant, it will be very interesting to provide and discuss what grants have been quoted more often- and in this sense have been more influential. 

 12.- Overall, It is. a pity that the paper provides such a shallow level of analysis. For example, the overall number of papers and citations, does not really help to understand the impact of ELIXIR or ELIXIR-IT.  Providing number without a context does not really advance the analysis of impact and legacy.

Is the rationale for developing the new method (or application) clearly explained?

Yes

Is the description of the method technically sound?

Partly

Are the conclusions about the method and its performance adequately supported by the findings presented in the article?

Partly

If any results are presented, are all the source data underlying the results available to ensure full reproducibility?

Partly

Are sufficient details provided to allow replication of the method development and its use by others?

No

Reviewer Expertise:

Regarding this article: NLP and text mining and active participant in ELIXIR.

I confirm that I have read this submission and believe that I have an appropriate level of expertise to confirm that it is of an acceptable scientific standard, however I have significant reservations, as outlined above.

# Reviewer report: Reviewer response for version 3

**DOI:** 10.5256/f1000research.180668.r379750

**Published:** 2025-05-10

**Authors:** Shuichi Kawashima

**Affiliations:** 1 Database Center for Life Science, Chiba, Japan

I thank the authors for their thorough revisions and clarifications. All of my previous concerns, including the issue regarding the citation count discrepancy, have now been addressed.

 I have no further comments.

 I recommend acceptance of the manuscript.

Is the rationale for developing the new method (or application) clearly explained?

Yes

Is the description of the method technically sound?

Yes

Are the conclusions about the method and its performance adequately supported by the findings presented in the article?

Yes

If any results are presented, are all the source data underlying the results available to ensure full reproducibility?

Partly

Are sufficient details provided to allow replication of the method development and its use by others?

Partly

Reviewer Expertise:

bioinformatics, semantic web, database

I confirm that I have read this submission and believe that I have an appropriate level of expertise to confirm that it is of an acceptable scientific standard.

# Reviewer report: Reviewer response for version 2

**DOI:** 10.5256/f1000research.178964.r375436

**Published:** 2025-04-14

**Authors:** Shuichi Kawashima

**Affiliations:** 1 Database Center for Life Science, Chiba, Japan

I appreciate the authors' efforts to revise the manuscript in response to my previous comments. Most of my concerns have been appropriately addressed, and the manuscript is significantly improved in terms of clarity and reproducibility.

 Remaining Issue – Citation Count Discrepancy (Figure 2 vs. Text):

 The authors state that they have resolved the discrepancy between Figure 2 and the corresponding citation count in the text. However, upon reviewing the current version of the manuscript and figure, I was unable to identify any changes from the originally submitted version. Both the text and Figure 2 appear unchanged, and the inconsistency (i.e., the text referring to ~5,000 citations vs. the figure showing ~6,721) remains unresolved. I would appreciate clarification on this point—specifically, whether an update was made but not reflected in the posted version, or whether the discrepancy remains due to another reason.

Is the rationale for developing the new method (or application) clearly explained?

Yes

Is the description of the method technically sound?

Yes

Are the conclusions about the method and its performance adequately supported by the findings presented in the article?

Yes

If any results are presented, are all the source data underlying the results available to ensure full reproducibility?

Partly

Are sufficient details provided to allow replication of the method development and its use by others?

Partly

Reviewer Expertise:

bioinformatics, semantic web, database

I confirm that I have read this submission and believe that I have an appropriate level of expertise to confirm that it is of an acceptable scientific standard, however I have significant reservations, as outlined above.

# Reviewer report: Reviewer response for version 1

**DOI:** 10.5256/f1000research.173833.r354532

**Published:** 2025-02-27

**Authors:** Shuichi Kawashima

**Affiliations:** 1 Database Center for Life Science, Chiba, Japan

This manuscript presents an approach to identifying and measuring the scientific impact of ELIXIR-supported publications, employing text-mining of EuropePMC alongside manual curation. It offers valuable insights into the output and influence of a distributed research infrastructure, with an in-depth case study of ELIXIR Italy. The methodology is generally sound and relevant; however, several clarifications are needed to ensure transparency and reproducibility.

**Major Concerns:**

 * The authors should include concrete examples (e.g., sample URLs or code snippets) illustrating how EuropePMC’s API was queried and how retrieved data were processed. This addition would strengthen reproducibility.

 * A subset of known ELIXIR-supported articles had to be manually whitelisted, suggesting certain limitations in the automated text-mining. The authors are encouraged to explain why these articles failed to appear in the initial searches (e.g., unusual acknowledgments or missing metadata), as such detail would be instructive for others employing similar methods.

**Minor concerns:**

 * The figure 2 indicates ~6,721 citations, whereas the text mentions 5,000. This discrepancy should be resolved for clarity.

 * According to the text, 408 unique publications were supported by ELIXIR Italy from 2015 to 2023. Figure 3 shows the distribution of these publications across the Node’s institutes. If one sums the values for all institutes in Figure 3, the total appears to be 772, which does not match 408. The authors should clarify whether Figure 3 double-counts papers co-authored by multiple institutes or if other factors account for the difference.

 The manuscript is well-structured, and the research offers a promising approach for tracking the impact of distributed infrastructures. Addressing the concerns above will strengthen the paper’s clarity and transparency. I recommend minor revision prior to acceptance.

Is the rationale for developing the new method (or application) clearly explained?

Yes

Is the description of the method technically sound?

Yes

Are the conclusions about the method and its performance adequately supported by the findings presented in the article?

Yes

If any results are presented, are all the source data underlying the results available to ensure full reproducibility?

Partly

Are sufficient details provided to allow replication of the method development and its use by others?

Partly

Reviewer Expertise:

bioinformatics, semantic web, database

I confirm that I have read this submission and believe that I have an appropriate level of expertise to confirm that it is of an acceptable scientific standard, however I have significant reservations, as outlined above.

# Reviewer report: Reviewer response for version 1

**DOI:** 10.5256/f1000research.173833.r354530

**Published:** 2025-01-06

**Authors:** Helen M. Berman

**Affiliations:** 1 Chemistry and Chemical Biology, Rutgers, The State University of New Jersey, Piscataway, NJ, USA

This article describes a method to assess the impact of ELIXIR supported research. This was done by using text mining to search for relevant articles contained in EuropePMC. This procedure was used for all ELIXIR nodes and for ELIXIR Italy. In the latter case the search terms were modified to be appropriate for that node. In the first step specific search terms were used to search sections of articles. This automated procedure was followed by manual curation.

 It is important to have quantitative methods to analyze impact of the ELIXIR infrastructure and this is a good first step. The paper is well written, and the limitations of the approach are adequately described. For example, the EuropePMC does not index all the journals that describe projects funded under the ELIXIR umbrella. It is also disturbing that some articles are published in journals that are behind paywalls.

 Although the curated datasets are put into Zenodo, the authors might consider making a website that would allow authors to submit and curate their ELIXIR related articles.

Is the rationale for developing the new method (or application) clearly explained?

Yes

Is the description of the method technically sound?

Yes

Are the conclusions about the method and its performance adequately supported by the findings presented in the article?

Yes

If any results are presented, are all the source data underlying the results available to ensure full reproducibility?

Yes

Are sufficient details provided to allow replication of the method development and its use by others?

Partly

Reviewer Expertise:

Structural bioinformatics, structural biology

I confirm that I have read this submission and believe that I have an appropriate level of expertise to confirm that it is of an acceptable scientific standard.
